# Supplementary material for: DMRforPairs: identifying Differentially Methylated Regions between unique samples using array based methylation profiles
Source: BMC Bioinformatics. 2014 May 15;15:141. doi: 10.1186/1471-2105-15-141 (PMC4046028; doi:10.1186/1471-2105-15-141)
Supplement: Additional file 2 — DMRforPairs output for the comparison of A431-MCF7 and NA17018-NA17105. Please start from the HTML files in each folder. Available via the BMC Bioinformatics website. [file 1471-2105-15-141-S2.zip › 1394847754114233_MOESM2_ESM/A431_MCF7/figures/17345.pdf]

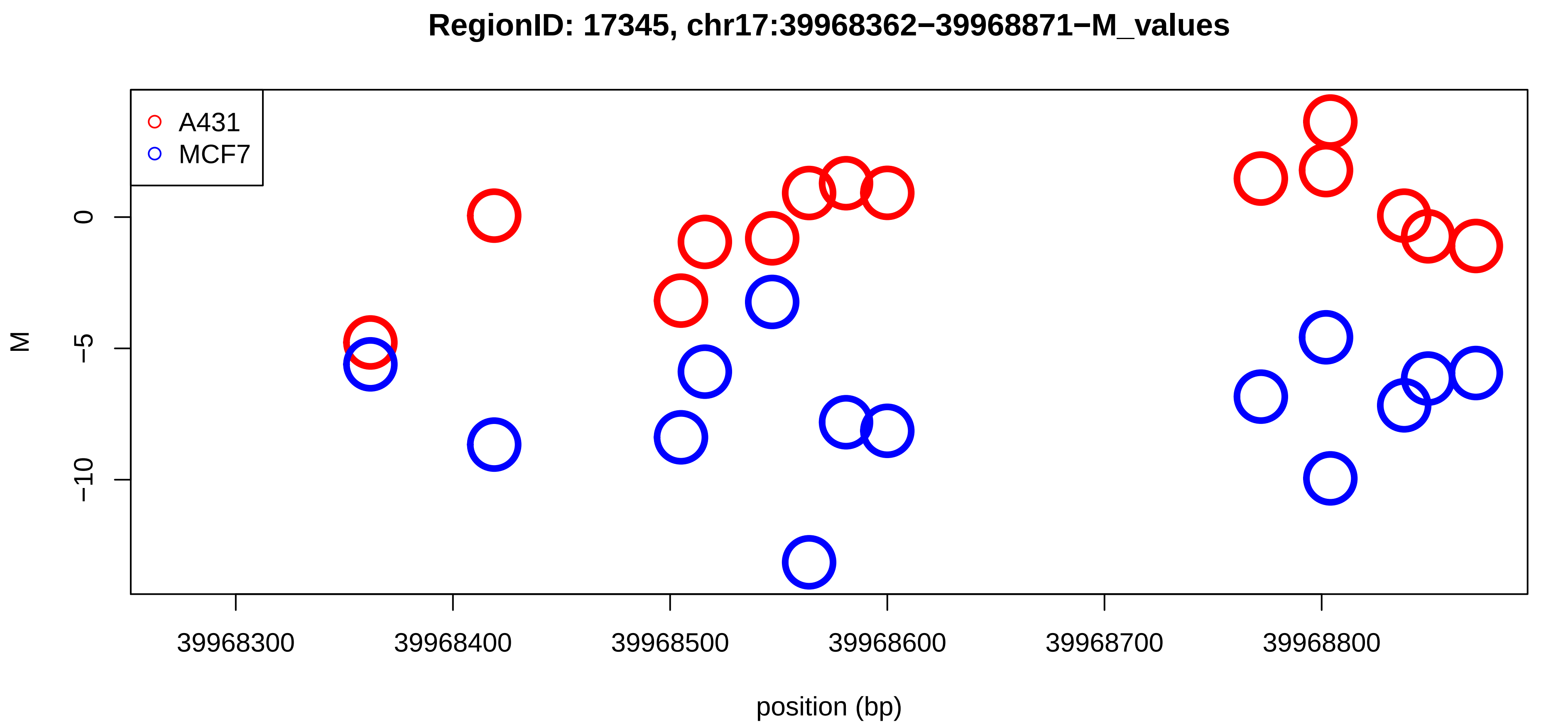

RegionID: 17345, chr17:39968362−39968871−M_values
A431
MCF7
M
0
−5
−10
39968300
39968400
39968500
39968600
39968700
39968800
position (bp)

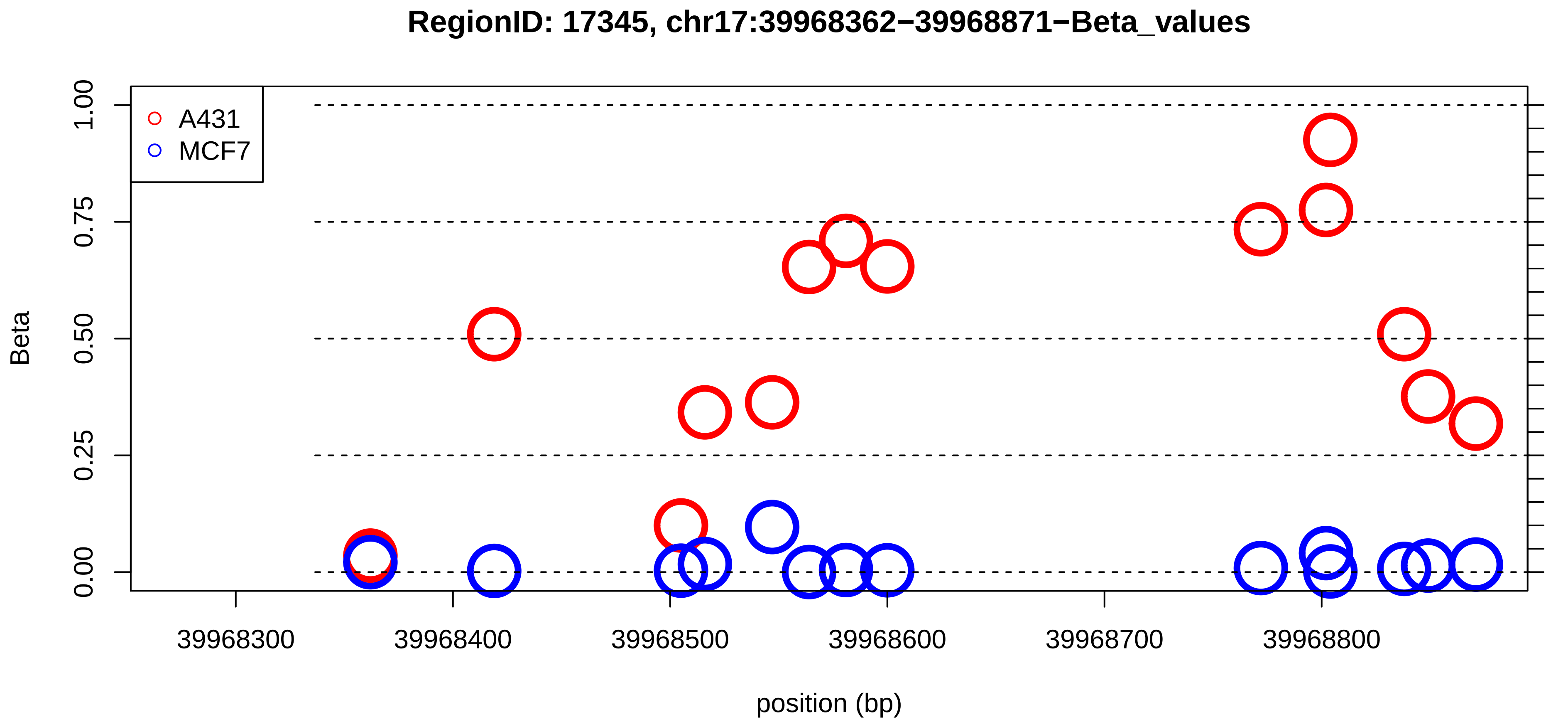

RegionID: 17345, chr17:39968362−39968871−Beta_values
A431
MCF7
Beta
0.00
0.25
0.50
0.75
1.00
39968300
39968400
39968500
39968600
39968700
39968800
position (bp)

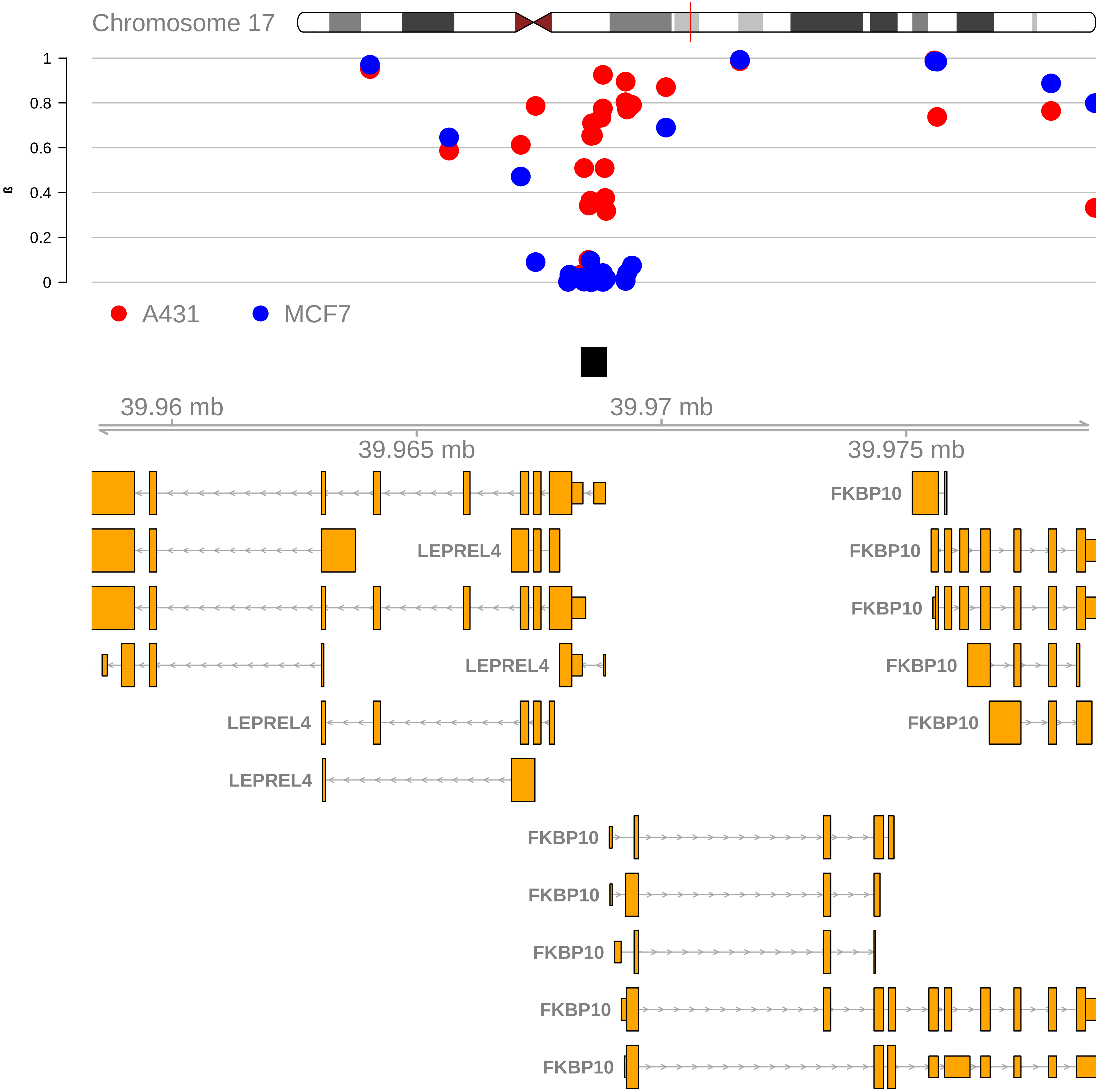

Chromosome 17
ß
1
0.8
0.6
0.4
0.2
0
A431
MCF7
39.96 mb
39.97 mb
39.965 mb
39.975 mb
FKBP10
LEPREL4
FKBP10
FKBP10
LEPREL4
FKBP10
LEPREL4
FKBP10
LEPREL4
FKBP10
FKBP10
FKBP10
FKBP10
FKBP10
